# Exploring the Influencing Factors of COVID-19 Vaccination Willingness among Young Adults in China

**DOI:** 10.3390/ijerph20053960

**Published:** 2023-02-23

**Authors:** Yue Su, Sijia Li, Feng Huang, Jia Xue, Tingshao Zhu

**Affiliations:** 1 CAS Key Laboratory of Behavioral Science, Institute of Psychology, Chinese Academy of Sciences, Beijing 100101, China; 2 Department of Psychology, University of Chinese Academy of Sciences, Beijing 100049, China; 3 Department of Social Work and Social Administration, The University of Hong Kong, Hong Kong; 4 Factor-Inwentash Faculty of Social Work, University of Toronto, Toronto, ON M5S 1A1, Canada; 5 Faculty of Information, University of Toronto, Toronto, ON M5S 1A1, Canada

**Keywords:** COVID-19 vaccine, vaccine intention, vaccine willingness, influencing factor, vaccine hesitancy, thematic analysis, topic model, qualitative research

## Abstract

Vaccine uptake is considered as one of the most effective methods of defending against COVID-19 (coronavirus disease 2019). However, many young adults are hesitant regarding COVID-19 vaccines, and they actually play an important role in virus transmission. Based on a multi-theory model, this study aims to explore the influencing factors related to COVID-19 vaccine willingness among young adults in China. Using semi-structured interviews, this study explored the factors that would motivate young adults with vaccine hesitancy to get the COVID-19 vaccine. Thematic analysis was used to analyze the interview data with topic modeling as a complementarity method. After comparing the differences and similarities of results generated by thematic analysis and topic modeling, this study ultimately identified ten key factors related to COVID-19 vaccination intention, including the effectiveness and safety of vaccines, application range of vaccine, etc. This study combined thematic analysis with machine learning and provided a comprehensive and nuanced picture of facilitating factors for COVID-19 vaccine uptake among Chinese young adults. Results may be taken as potential themes for authorities and public health workers in vaccination campaigns.

## 1. Introduction

Since the outbreak of the COVID-19 (coronavirus disease 2019) pandemic in December 2019, nearly every country worldwide has suffered greatly over a long period of time. To combat COVID-19, the general public, professionals, and authorities have made tremendous efforts and devised multiple strategies to prevent the transmission of SARS-CoV-2 [1]. Among these strategies, vaccination is considered as one of the most effective methods to control the pandemic [2].

However, many people are hesitant regarding the COVID-19 vaccine. SAGE (The Strategic Advisory Group of Experts on Immunization) defined vaccine hesitancy as a “delay in acceptance or refusal of vaccines despite availability of vaccine services” [3]. Vaccine hesitancy has been identified as one of the ten greatest threats to public health by the World Health Organization [4]. Therefore, exploring and identifying factors that influence people to vaccinate against COVID-19 are high-priority tasks [5]. 

Young adults are a significant group to be concentrated on for the promotion of immunization campaign. Young people are vulnerable to coronavirus infection due to jobs and campus life [6]. Moreover, the young population plays an important role in virus transmission given the fact that they have high rates of asymptomatic infections and have active social lives [7]. Besides, the period of young adulthood experiences changes in personal values and beliefs [8], which may have influences on the adherence to preventive behaviors [9]. Considering the high frequency of social media use among young people and their tendency to seek health information online, their vaccine attitudes may be easily affected by social influences [10]. Furthermore, young adults could also work as potential change agents and promote safer behaviors within their families and communities [11]. In this study, we aim to explore influencing factors related to COVID-19 vaccine willingness among young adults in China.

Many factors that would influence the COVID-19 vaccination intention of young adults were identified, such as concerns about vaccine safety [12,13,14], side effects [11,12], effectiveness [11,13,15], political concerns [11,14], thinking others are in greater need of the vaccine [12,15,16], and social support [8]. Researchers conducted a cross-sectional survey among Chinese college students and found that participants who believed in vaccine safety and effectiveness in preventing infection were more likely to accept the COVID-19 vaccine [17]. Besides, a cross-sectional study, based on the extended theory of planned behavior, surveyed mainland Chinese university students and reported that knowledge about the COVID-19 vaccine as well as the risk perception of COVID-19 were main factors influencing COVID-19 vaccine attitude [18]. Moreover, a study based on the health belief model conducted interviews among young adults in China and proposed that social benefits and worry reduction were positive factors of vaccination intention [19]. Other factors for Chinese young adults included worrying about adverse effects [20], vaccine safety [17], family support [21], and attitudes of surrounding individuals [22]. Although there were many studies on COVID-19 vaccination willingness, the subjective experience of young adults was insufficient in research. Many studies used questionnaires to survey individuals, and participants may not explicate the reasons of their responses in detail in most cases. Exploring the experiences and opinions of young adults in an explicit and flexible way may further the understanding of vaccination intention. Furthermore, semi-structured interviews may provide participants the opportunity to adequately express their responses using their own narratives. In addition to the common qualitative analysis methods, we propose that multiple analysis methods may help identifying potential promising factors from different viewpoints. From the perspective of young adults, this study aims at exploring lived experiences related to COVID-19 vaccination intention.

Researchers described health behavior as “any activity undertaken by a person believing himself to be healthy, for the purpose of preventing disease or detecting it in an asymptomatic stage” [23]. Furthermore, getting vaccinated could be considered as health behavior, which is also acknowledged in many relevant studies [24,25,26]. A theory related to health behaviors is needed to identify factors affecting vaccination in this study. Multi-theory model (MTM) is a fourth-generation theory explaining changes in health behaviors, which includes malleable constructs and can provide insights for both one-time and long-term behavioral changes [27]. MTM incorporates multiple theoretical perspectives and could work at individual, group, and community levels [27]. Considering vaccination uptake is a complex issue involving multiple factors and complicated contexts, MTM could be used to explore such a phenomenon from a comprehensive and extensive perspective. MTM consists of two components: initiation and sustenance, which differentiates between these two components in a detailed way and provides a nuanced understanding of health behaviors. Since vaccination differs from long-term behaviors, such as physical activity, we have chosen to focus on the first component in our study. Initiation is explained in terms of three constructs: participatory dialogue, behavioral confidence, and changes in the physical environment [27]. MTM has been used by previous studies to explain health behaviors changes. For instance, a cross-sectional study based on MTM explored COVID-19 booster vaccination hesitancy in the United States and found that the hesitant group had lower odds of behavioral confidence than the non-hesitant group [28]. Researchers also used MTM to explain the handwashing behavior in an American sample of college students during the COVID-19 pandemic [29] as well as the intentions of physical activity behavior among Chinese pregnant women [30]. In the present study, we proposed to use this framework to explore COVID-19 vaccination intentions in China.

With MTM working as the theoretical basis, this study focused on the subjective experience of individuals and explored what factors might be able to tip the scales toward a decision among young adults in China who are initially hesitant to get vaccinated. Specifically, we conduct semi-structured interviews for data collection and employ qualitative thematic analysis and machine learning approach to analyze data.

## 2. Materials and Methods

This study used semi-structured interviews to explore the lived experiences of participants and to ensure the in-depth communication of concerns and motivations related to COVID-19 vaccination uptake among young adults. To understand the interview results in a more comprehensive and nuanced way, multiple methods were used in this study to analyze the interview data, including thematic analysis and topic modeling. Thematic analysis is commonly used when identifying main themes [31].Moreover, topic modeling, as an unsupervised machine learning method, could categorize clusters and identify common topics in texts [32]. After that, we examined and compared the findings of these two methods to obtain a final set of influencing factors related to COVID-19 vaccination intention.

The interview guide was developed with reference to the previous literature on the perceptions of vaccination [33,34,35]. Four experts with expertise in COVID-19 and the COVID-19 vaccine agreed on the contents of the interview guide following in-depth discussion and analysis. This interview guide contained two sections: one section pertaining to the concerns that people may have regarding a vaccine, and the other section regarding the information and factors that people considered to be influential when discussing COVID-19 vaccine uptake.

### 2.1. Participant Recruitment

Individuals who met the following criteria were eligible to participate in this research. They should: (1) be mainland Chinese residents, (2) young adults aged between 18 and 35 years old, (3) be people who were self-reported to be hesitant about the COVID-19 vaccine and then changed their attitudes, and (4) be fluent in Chinese when communicating with others. Recruitment advertisements were posted on social media by researchers, and people who met the requirements and who were interested in this interview completed the questionnaire contained in the advertisement and included their phone number and a time in which they would be available. Purposive sampling method was used to assist with participants screening to balance gender distribution and enrich the regional distribution. The participants were compensated with a cash reward in gratitude for their time and contribution. Our study was approved in advance by the Ethics Committee of the Institute of Psychology, Chinese Academy of Sciences (approval number: H15009).

### 2.2. Data Collection

All interviews were performed via the Tencent Meeting (Tencent Meeting, version 3.8.4.417; Tencent Holdings Ltd.: Shenzhen, China, 2021), which is a remote meeting application that provides cloud video- and audio-conferencing services. The research assistants majoring in psychology with interviewing expertise conducted these interviews with guidance from the principal researchers. These interviews lasted approximately 30–60 min. Informed consent was obtained from each participant. The interviews were audio-recorded for further analysis. The data collection ended and the sample size was confirmed when data saturation for the themes in framework was reached in our sample after team discussions [36]. Subsequently, the audio recordings were transcribed verbatim by the interviewers to produce the interview transcripts.

### 2.3. Data Analysis

#### 2.3.1. Thematic Analysis

Thematic analysis is a traditional qualitative method that is widely used in the health sciences [37]. Based on the interview transcripts, we performed thematic analysis to code interview data manually. In this research, we followed the thematic analysis steps suggested by Braun and Clarke [37], which included familiarizing ourselves with our data, generating initial codes, searching for themes, reviewing themes, defining and naming themes, and producing the report. The analysis of transcribed interviews was guided by MTM framework. To enhance the rigor of the analysis, the initial code development and subsequent theme generation and definition were discussed between two researchers (the first author and the second author) until a final agreement was reached [38]. To demonstrate the meanings of the themes more clearly, we selected concrete examples that participants expressed to characterize each theme.

However, such qualitative studies are also considered to rely on the understandings of individuals [39]. Moreover, when the time required for interviews is long and the corresponding transcripts are large, repeated reading and examination of transcripts and multiple rounds of discussions among researchers can consume a great deal of cognitive resources. Considering the long periods of interviews in this study could increase the possibility of oversights occurrence, we used topic modeling to complement thematic analysis and provide insights from a novel perspective.

#### 2.3.2. Topic Modeling

Topic modeling has been widely used in text analysis [40,41]. Topic modeling can provide multiple word clusters as the main topics for understanding the basic and underlying dimensions of linguistic data [42]. Topic modeling has been used in many fields, such as social media analysis [43] and couple therapy trials [42]. In this study, we used topic modeling to supplement our interview transcript analysis. By combining thematic analysis and topic modeling, we may provide a novel and comprehensive perspective to facilitate the investigation of vaccination intention among young adults.

We collected all sets of words spoken by participants contained in interview transcripts as participant response texts. These texts were appropriate materials for topic modeling. First, we used Python (Python 3, version 3.8.8; Python Software Foundation: Wilmington, Delaware, United States, 2021) to preprocess all texts to ensure the quality and interpretability of the analysis results. The preprocessing steps included the elimination of special symbols, word segmentation, and word elimination (i.e., stop words and words whose length was less than two characters were removed). Such word elimination can reduce the occurrence of irrelevant terms and noises and thus enhance the performance of the topic model algorithm [44].

We thus obtained the final dataset for topic modeling. Subsequently, we used the latent Dirichlet allocation (LDA) algorithm to explore the main topics and structures contained in the dataset. LDA is a common method for topic modeling and is frequently used to investigate multiple topics within a set of documents [43,45]. LDA is a probabilistic model and can facilitate the inference of latent thematic structures from documents without prior knowledge or manual labels [44]. The Mallet version of LDA is implemented in the Python package Gensim and is considered to perform better than LDA [46]. Therefore, we used the Mallet version of LDA to construct the topic model and to investigate the thematic patterns in our data.

The number of topics is an important parameter for the model performance. To determine the appropriate number of topics, we tried different numbers of topics that ranged from two to thirty and calculated the coherence score for each model. It is advisable to use a coherence score in the measurement of model performance. A higher coherence score indicates a more precise model [44]. Based on the coherence score, the most appropriate number of topics was chosen and the final model of topics was confirmed. Subsequently, we employed pyLDAvis to visualize the topic model result. PyLDAvis is a Python package that provides a visual interface to conceptualize inter-topic distance [47].

The final step of topic modeling was to analyze, identify, and describe themes in accordance with the results of the LDA algorithm. After identifying the number of topics suggested by the coherence score, two authors discussed the top twenty keywords for each topic and reviewed the corresponding expressions in interview transcripts. Subsequently, they reached an agreement concerning a label and description for each theme.

## 3. Results

In this study, we carried out 12 interviews. Table 1 showed the demographic information of our interview participants. The gender distribution of participants reached a balanced gender ratio. Besides, the regional distribution was rather diverse.

### 3.1. Thematic Analysis Results

After concluding all interviews, we used three constructs in MTM as a framework and conducted thematic analysis to analyze interview transcripts. Thirty-one initial codes were generated (for more details, please refer to the Appendix A Table A1) and nine key factors were identified. More specifically, we identified four key factors belonging to participatory dialogue, one key factor that was closely related to behavioral confidence, and two key factors pertaining to changes in the physical environment. Besides, we found two novel key factors beyond MTM. The final interview results are shown in Table 2.

Construct 1: Participatory Dialogue

The main content of participatory dialogue focused on the advantages and disadvantages of changes in health behavior [27], including the *effectiveness and safety of vaccines, scientific and objective expression, perceived benefits,* and *social responsibility*.

The first factor is the *effectiveness and safety of vaccines*. This factor emphasized concerns regarding whether the vaccine would protect against COVID-19 effectively or whether it would pose a threat to personal safety. Participants reported that they were worried about the side effects of vaccination. Some participants wanted to know more about why some people were not suitable for vaccination.

*Scientific and objective expression* emphasized the fact that vaccine-related information should avoid the inclusion of arbitrary or ambiguous expressions, provide a comprehensive source of vaccination information from an objective perspective, and use accurate and easy-to-understand language. Some participants noted that they had heard that the effectiveness rate of COVID-19 vaccines in Brazil was low, and they believed that it was rather honest to publish such information instead of merely praising the vaccine.

*Perceived benefits* referred to the benefits perceived by individuals following vaccination. For example, participants noted that they would get vaccinated actively if doing so made it easier to enter and leave campus.

*Social responsibility* entails that people are aware, feel, and believe that an individual should be responsible for others, i.e., his or her family, group, country, and society. Moreover, such a person should also have the corresponding conscious attitude that an individual should abide by norms, accept responsibility, and fulfill obligations. Some participants indicated that vaccination uptake is a sign of being responsible to others and to society.

Our interview results identified this construct. *The effectiveness and safety of vaccines* and *scientific and objective expression* both focused on the disadvantages that people may face when considering vaccination uptake, such as side effects and limited efficacy. The *perceived benefits* emphasized the advantages perceived by individuals, while *social responsibility* underlined the advantages shared by the community and society.

Construct 2: Behavioral Confidence

Behavioral confidence emphasized the certainty of individuals when performing a certain behavior, such as receiving the COVID-19 vaccine. The source of behavioral confidence can come from external sources. For instance, people would refer to the opinions of influential people in real life [27]. In this study, we confirmed one key factor, *External reference,* in this construct.

*External reference* referred to situations in which an individual intended to engage in a certain behavior or develop a certain attitude by using a specific individual or group as a reference and object of comparison. These reference objects included specific social groups, the majority of people in the society, people surrounding the individual in question, and people from different political and cultural backgrounds. Some participants noted that the ways in which people around them viewed vaccines were important for them. If close friends and family chose to become vaccinated, these participants would also approve of the vaccine.

Construct 3: Changes in the physical environment

Changes in the physical environment emphasized the importance of individuals’ physical environment, including the availability, accessibility, and convenience of resources, which could also affect behavioral intentions. In this study, this construct contained two key factors, i.e., *the convenience of vaccines* and *the perceived necessity and urgency of vaccination uptake.*

*The convenience of vaccines* referred to the costs that individuals would be required to consider and pay during the process of vaccination, such as costs related to distance and time. Participants noted that if there were many vaccination sites and if it were convenient to get vaccinated, they would be more willing to receive the COVID-19 vaccine.

*The perceived necessity and urgency of vaccination uptake* emphasized the degree of intensity and urgency associated with the need for vaccination that individuals experienced in the context of the current social and epidemic situation. Several participants noted that if it were difficult to make a vaccination appointment or if the vaccine were scarce, they would be more willing to receive the vaccine.

In this study, we found that although other key factors cannot simply be integrated into MTM constructs, they were also important influencing factors related to COVID-19 vaccination intention. These factors included *perceived threat to the freedom of choosing getting vaccinated* and *policy requirements*.

*Perceived threat to the freedom of choosing getting vaccinated* referred to situations in which people felt some degree of threat to their freedom when certain acts aimed at persuasion interfered with individual free choice. Some participants indicated that many reports claimed that people must be vaccinated instead of that they should be vaccinated. This kind of expression made these participants more resistant to receiving the COVID-19 vaccine.

*Policy requirements* entailed that an individual would abandon his or her own opinion and behave in accordance with policy demands and group regulations. Some participants indicated that if the relevant authorities implemented mandatory vaccination against COVID-19, they would obey this requirement and become vaccinated.

### 3.2. Topic Modeling Results

**Figure 1:**
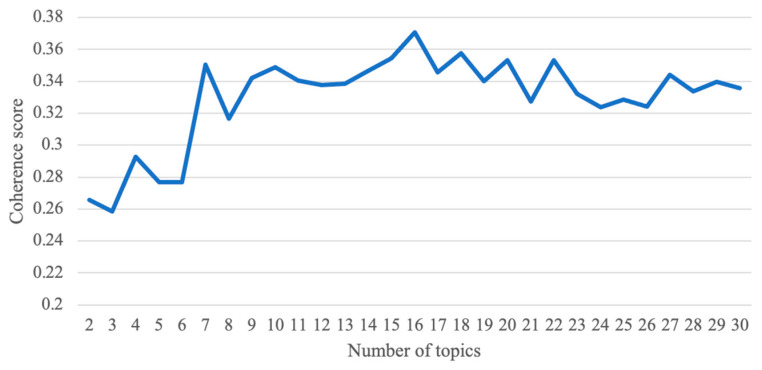
The coherence score results.

To determine the appropriate number of topics for topic modeling, we calculated the coherence score for each topic model. Figure 1 presents the coherence score result for each topic model, which was developed based on a different number of topics. We finally chose seven as the most appropriate number of topics for topic modeling based on two considerations. First, when the number of topics was set to seven, the coherence scores were high, thus indicating the good performance of the topic model. Second, our corpus was not very large, and too many topics could dilute the main focus of our text materials. Thus, in comparison with other numbers of topics that exhibited rather high coherence scores, such as sixteen and eighteen, seven contained the important topics to the greatest possible degree, on the one hand, and avoided the risk of confusing the main focus of the study, on the other [48].

**Figure 2:**
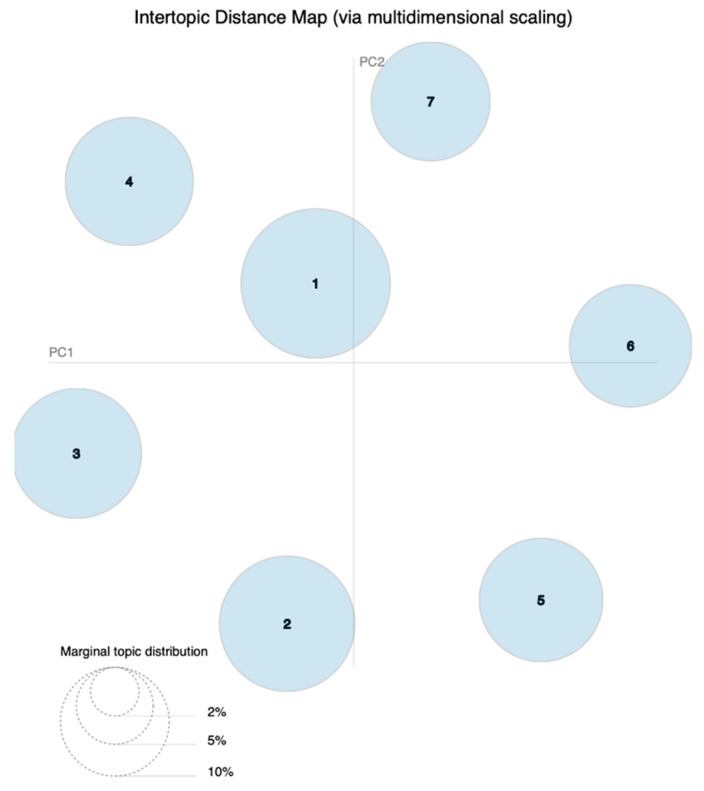
The inter-topic distance map.

The inter-topic distance map is shown in Figure 2. Each bubble in the inter-topic distance map represents a topic, and the area of the bubble indicates the prevalence of this topic [49]. The distances of topics are scaled multidimensionally onto two axes [50]. Relatively large and nonoverlapping bubbles in the map suggest a proper topic model result [49]. Each bubble represents a different topic ranging from Topic 1 to Topic 7 in our study. The bubbles shown in Figure 2 are relatively large and do not overlap, thus confirming the reasonability of using seven topics and suggesting that this approach produces a good topic model. The topics are ordered in descending order of prevalence in the corpus [49].

Table 3 shows the results of the topic model including seven topics and the top twenty relevant words for each topic in the corpus. The numbers of topics are in accordance with the numbers shown in the inter-topic distance map.

Following the analysis and discussion of the seven topics grouped by topic modeling, we identified these seven topics as our final topic categories and reached an agreement concerning a label for each topic. This result is shown in Table 4.

The first topic (Topic #1) was called *influences from others’ experiences*. This topic focused on the fact that people refer to the experiences and behaviors of other people, such as friends and colleagues, when making decisions regarding vaccination uptake. The feedback of others was of great value to these people.

The second topic (Topic #2) was labeled *potential costs during the vaccination process*. This topic included two aspects. One such aspect was the social pressure that people might experience if they remained unvaccinated. The other aspect pertained to the time and energy costs that people would pay if they were to consider becoming vaccinated. In other words, this topic concerned all the costs that people should consider when making a vaccination plan.

The third topic (Topic #3) was characterized as the *side effects of the COVID-19 vaccine*. This topic focused on concerns regarding the side effects of the vaccines. More specifically, this topic emphasized how people’s bodies would feel after becoming vaccinated, which caused them to worry.

The fourth topic (Topic #4) was labeled *application range of vaccine*. This topic focused on the application population and situations, that is, the kinds of people that were able to become vaccinated, the kinds of people that should wait, the kinds of people that should not receive a vaccination, and the situations in which vaccination would be useful, as well as those in which full vaccination would not be sufficient, thus requiring other epidemic protection approaches (e.g., a nucleic acid test).

The fifth topic (Topic #5) was defined as *direct and transparent information*. This topic emphasized the fact that the language and expressions used in relation to the vaccine should be unambiguous, direct, sincere, and transparent.

The sixth topic (Topic #6) was termed *positive perceptions of the epidemic situation*. This topic referred to the necessity of vaccination uptake, and when people felt positive regarding their living circumstances and believed that their risk of infection was low, they noted that they might be less willing to become vaccinated.

The seventh topic (Topic #7) was called *safe and reliable information related to the COVID-19 vaccine*. This topic emphasized people’s attention to trustworthy information related to vaccine safety. More specifically, official media, medical professionals, and scientific reports that supported and verified vaccine safety would have some degree of impact on certain people.

### 3.3. Influencing Factors Related to COVID-19 Vaccination Intention

From a practical perspective, we want to focus on the factors that have the greatest practical value in the context of vaccination promotion, and they can be divided into two categories: nonmanipulable and manipulable factors. Nonmanipulable factors refer to factors that are directly related to the COVID-19 vaccine itself, such as experimental results and objective data. Manipulable factors refer to factors that are indirectly related to the COVID-19 vaccine and commonly focus on external circumstances rather than information pertaining to the vaccine per se. It is rather easy for researchers to change the presentation of these factors in practice as a means of influencing the feelings of individuals.

Our two data analysis approaches used in this study led to two similar but subtly different results. Table 5 shows the detailed findings of each analysis method.

With respect to the theme “nonmanipulable factors”, the thematic analysis identified one key factor, *the effectiveness and safety of vaccines*. The topic model identified two key factors, i.e., *the side effects of the COVID-19 vaccine* and *application range of vaccine*. We found that topic modeling confirmed more factors than thematic analysis. Moreover, *the effectiveness and safety of vaccines* and *the side effects of the COVID-19 vaccine* shared a partially similar focus, as the safety referenced by the former factor and the side effects noted by the latter factor both emphasized the perceived threat of vaccines and the damage that they can cause. However, *application range of vaccine* emphasized the relevant situation and the groups to which vaccines should or should not apply. This factor indicated a semantic connection between the targeted group and the situation of use, which differed from a concentration on the *effectiveness and safety of vaccines* and indeed supplied a new perspective concerning nonmanipulable factors.

As manipulable factors, the thematic analysis identified eight key factors, that is, convenience of the vaccine, social responsibility, external reference, perceived necessity and urgency of vaccination uptake, perceived threat to the freedom of choosing getting vaccinated, perceived benefits, scientific and objective expression, and policy requirements. The topic model identified five key factors: influences from others’ experiences, potential costs during the vaccination process, direct and transparent information, positive perceptions of the epidemic situation, and safe and reliable information related to the COVID-19 vaccine.

According to the meanings of these factors discussed above, we must acknowledge that *influences from others’ experiences* were quite similar to *external reference*, both of which highlighted influences from external sources. In addition, *direct and transparent information* and *scientific and objective expression* shared a common focus on the unambiguous and accurate language used to convey vaccine-related information. Moreover, *potential costs during the vaccination process* were also identified in the results of thematic analysis. In this topic, the first aspect, i.e., social pressure, was categorized as part of the *external reference* category in the thematic analysis. The second aspect, time and energy costs, was quite similar to *the convenience of the vaccine* emphasized by the thematic analysis. We also suggested that the *safe and reliable information related to the COVID-19 vaccine* factor found by the topic modeling was part of the *external reference* category in the thematic analysis. The former topic highlighted the importance of having reliable information sources, such as medical professionals, which was related to the latter factor.

In addition, one interesting finding was that the same factor was found by these two analysis results, but was presented in different directions between these two cases. *The perceived necessity and urgency of vaccination uptake* emphasized the fact that pressure and risks resulting from situations could cause people to feel a sense of urgency and to become more active in receiving the vaccination. Meanwhile, *the positive perception of an epidemic situation* highlighted the fact that a positive feeling toward the epidemic could decrease the public’s willingness to become vaccinated. Regardless of the direction, these two factors both pertained to the necessity of vaccine uptake.

In addition, the thematic analysis found *social responsibility, perceived threat to freedom of choosing getting vaccinated, perceived benefits,* and *policy requirements*, which were not included in the topic modeling results. These findings suggested that with respect to manipulable factors, the thematic analysis provided more detailed and distinct results compared to topic modeling.

Taking the results of both the thematic analysis and the topic modeling into consideration, we added *application range of vaccine* to the list of nonmanipulable factors based on the thematic analysis, and ultimately identified ten key factors related to COVID-19 vaccination intention.

## 4. Discussion

This study explored influencing factors related to public vaccination willingness based on semi-structured interviews. Following data collection, we employed two approaches to analyze the transcribed interviews. Using thematic analysis to code the transcripts manually, we identified seven key factors pertaining to the MTM constructs and included two novel influencing factors as supplements. We also used the topic modeling method LDA to examine the semantic pattern of the transcripts and thus identified seven key factors. Combining these two results, we analyzed the similarities and differences between them and confirmed ten influencing factors as a final set.

Some factors in this study have also been identified in previous research. In a systematic review by Wake, perceived risk of COVID-19, norms, the perceived benefits of the vaccine, the perceived efficacy of the COVID-19 vaccination, and COVID-19 vaccine safety concerns were found to be associated with COVID-19 vaccine uptake willingness [51], thus confirming the rationality of our results. We suggest that these influencing factors may be effective generally for COVID-19 vaccine campaign. For mass immunization campaign, these factors may work as the focus of vaccine communication in the long term.

However, several factors identified by previous studies did not emerge in our study. Liu and Liu found that in the category of physical opportunities, monetary concerns could lead to the refusal of the vaccine [52]. We did not find any factors related to affordability in our study. When the vaccine campaign began and the policy of free vaccinations was clearly announced [53], some predetermined factors were no longer suitable to the new situation.

Besides, the role of choice of freedom among Chinese young adults deserved the attention in this study. A cross-sectional study conducted in Italy suggested that believing in the freedom to choose whether to vaccinate was the key predictor of people with vaccine hesitancy [54]. However, in this study, we proposed that the perceived threat to freedom of choice may decrease the vaccination intention, and the perception of choice of freedom may be a motivating factor for immunization promotion. A previous study shared a similar idea that mandatory vaccination by employers would make people less likely to accept a COVID-19 vaccine [55]. Our study further explored such an idea by providing narratives of participants and emphasized the importance of the role of freedom of choice. 

This study used thematic analysis to analyze the interview data and employed topic modeling to supplement results. Our findings suggested that it is feasible to use thematic analysis as supplemented by topic modeling. In most cases, thematic analysis can provide detailed insights. The topics noted by the topic model were relatively rudimentary. However, topic modeling, as a method based on machine learning, can prevent oversights on the part of human beings facing high workloads and provide a novel perspective. According to our results, topic modeling indeed allowed us to identify some fresh relationships. Therefore, we suggest that future research should consider the addition of topic modeling to the thematic analysis to produce comprehensive analysis results.

Our study confirmed that MTM could function as a theoretical framework and explain influencing factors related to vaccination intention in China. Apart from the constructs associated with MTM, we also suggested novel factors as supplements. Our findings could provide nuanced understandings of related factors that can influence public COVID-19 vaccine uptake intention for young adults in China. By adopting semi-structured interviews, this study included the reflections and elaborations of participants sensitively and provided an insightful picture of effective factors related to COVID-19 vaccine hesitancy. Results might assist in determining the potential topics for targeted vaccination promotion programs. We suggest that authorities and public health workers may focus on one or several factors in accordance with the specific social situation and epidemic condition. For example, if the COVID-19 outbreak started in a country in close proximity to our own, authorities might consider emphasizing the severity of the disease and the risks faced by this country. Young people may thus feel the proximity of COVID-19, and their awareness of vaccination may be raised.

Our study faces certain limitations. First, our study was conducted in China, and our identification of influencing factors related to COVID-19 vaccine intention should thus be confined to China. People should use the results of this study with caution in the context of different cultural backgrounds. Besides, our study focused on the perspective of young adults. Little is known about whether these influencing factors found in this study would work well for the aged. Future study should further investigate the views of elderly people as well as other age groups. Further examination across generations may provide us with detailed information related to attitude changes concerning COVID-19 vaccine uptake. In addition, we suggest future researchers move forward to examine MTM framework and influencing factors confirmed in this study in a larger sample using epidemiological quantitative methods, which could expand the breadth of the framework and our findings. Besides, the differences of vaccination beliefs among different regions are worth being investigated in the future work. Although we made efforts to enrich the regional distribution, it is hard to derive the significant results due to the limited sample size in this study. We intend to further explore differences of effective influencing factors associated with COVID-19 on a regional level in a future study. 

## 5. Conclusions

This study aimed to explore influencing factors which would influence COVID-19 vaccination intention effectively for young people in China. We used semi-structured interviews to collect in-depth thoughts of participants and two analysis approaches (thematic analysis and topic modeling) to analyze the interview results. In conclusion, we identified ten different key factors in this study. The results suggested that multiple analysis approaches might perform well when analyzing interview data and could provide comprehensive perspectives and nuanced understandings. We suggest that authorities and public health workers may consider the key factors proposed by this study as potential themes when conducting a targeted vaccination promotion program.

## Figures and Tables

**Table 1:** Demographic information of interview participants.

Characteristics	*n* = 12
**Age** (Mean ± SD)	25.83 ± 2.98
**Gender***n* (%)	
Male	6 (50%)
Female	6 (50%)
**Education level***n* (%)	
College (three-year or two-year college diploma)	1 (8%)
Bachelors and above	11 (92%)
**Location**	
Eastern China	5 (41.67%)
Central China	4 (33.33%)
Western China	3 (25.00%)

**Table 2:** The results of thematic analysis.

The Constructs, Key Factors, and Codes
**Participatory dialogue**
*Effectiveness and safety of vaccines*
Vaccine safety, The restriction of vaccination uptake, Introduction of vaccine general information, Specific explanation of vaccination requirement, Data exhibition, Vaccine effectiveness, Providing a comparison
*Scientific and objective expression*
Dual role persuasion, Certainty of vaccination information, Popular science
*Perceived benefits*
Vaccination certification, Personal involvement degree, Extra benefits
*Social responsibility*
Social responsibility
**Behavioral confidence**
*External reference*
Group identification, Conformity, Reference group, In-group pressure, Psychological distance, Vicarious experience, Specialization, Credibility, Attraction, Official position, Unofficial position
**Changes in the physical environment**
*Convenience of vaccine*
Convenience of vaccine
*Perceived necessity and urgency of vaccination uptake*
Risk perception, Scarcity, Deadline strategy
**Beyond MTM**
*Perceived threat* *to the freedom of choosing getting vaccinated*
Perceived threat to the freedom of choosing getting vaccinated
*Policy requirement*
Policy requirement

**Table 3:** The topic model result with seven topics and the relevant words.

Topic	The Top 20 Relevant Word
1	Vaccine, get vaccinated, nation, attitude, around, information, promote, COVID-19, lots of, friend, matter, others, basically, really, job, official, after all, China, wait-and-see, several
2	Vaccine, that sort of, school, a little, popular science, community, two-shot, WeChat Moments, do not get vaccinated, is there any, influence, reason, time, first shot, second shot, three-shot, inconvenient, social, one-shot, life
3	Get vaccinated, side effect, school, thing, classmate, do not want to, matter, do not get vaccinated, body, suggestion, doctor, video, mentality, definitely, in this regard, six months, a few days, domestic, willingness, fear
4	Get vaccinated, definitely, influence, COVID-19 vaccine, adverse reactions, nation, concerns, a crowd of people, if, worry, epidemic, place, nucleic acid, a lot of, infect, allergy, child, impression, on earth, on the internet
5	Information, influence, focus on, policy, particularly, Weibo, I will, said to be, publicity, research and development, thing, precautions, like this, sometimes, effect, increase, remember, because of, observe, video
6	feel like, a little, news, kind of, epidemic, for, concerns, popular, situation, accept, particularly, time, infect, not quite, antibody, free, national people, less than, Douyin (Douyin, version 15.4.0; Beijing Microlive Vision Technology Co., Ltd.: Beijing, China, 2021), effect
7	Get vaccinated, vaccine, media, America, lots of, safety, probably, reports, temporary, be from, factors, data, scientific, appointment, tell, authority, indeed, this piece of, risk, the vaccination rate

**Table 4:** Seven topics produced by LDA with labeled names and examples.

Topics	Examples
Topic #1 *Influences from others’ experiences*	And the feelings of the people around me about being vaccinated will be more convincing for me. (Interview #2)
	(I) want to wait first and see the reactions of those who have already gotten vaccinated. (Interview #6)
Topic #2 *Potential costs during the vaccination process*	Even if he goes to the vaccination site for vaccination, it is still possible to fail to get vaccinated, and he has to wait for the second shot after getting the first shot, and sometimes the third shot is also required. He may feel it is not worthy to cost such time. (Interview #4)
	There is a piece of news, for example, that vaccines will be delivered to your home or your community, and time to get vaccinated is within a reasonable period, such as just going downstairs or walking for a few minutes. The vaccination process is very fast and convenient. Such a situation can also make me more willing (to get vaccinated). (Interview #11)
Topic #3 *The side effects of the COVID-19 vaccine*	Some people, I think, may be a little scarred psychologically. They are uncertain about this vaccine. Because when we published the information about this vaccine, we mentioned that this vaccine will cause some unpleasant symptoms, such as skin redness, fever, fatigue, nausea, and headaches, and then they might feel a little scared. (Interview #10)
	Another point may be related to doctors, such as the video channels or Douyin accounts of doctors. If doctors post videos to further explain the effects of the COVID-19 vaccine, as well as side effects and adverse reactions, and if they demonstrate such information very clearly, …… I think I will definitely be willing to get vaccinated. (Interview #5)
Topic #4 *Application range of vaccine*	There are some individuals with uncommon constitutions, such as suffering from allergies and purpura before. Can this group, on earth, get this vaccine? Some people asked me, “My child has had purpura before, can he get vaccinated? His school asks him to get vaccinated, can he?” Then I’m not very clear about this. (Interview #8)
	In fact, I still don’t understand why we need to do a nucleic acid test once a week even after vaccine uptake? All our hospitalized patients need nucleic acid tests, but some family members did not cooperate with us. They claimed that they have been vaccinated, so they didn’t need a nucleic acid test. (Interview #8)
Topic #5 *Direct and transparent information*	If I can see a very authoritative popular science document that can make people like me who are not willing to think about things easy to understand, then I would feel I understand it and consider getting vaccinated. Especially for the distinctions among different types of vaccines, the period that vaccines are effective, and the precautions of vaccination, the popular science document should explain these aspects clearly and concisely. (Interview #4)
	Take the lecture that I just mentioned as an example, the content of the lecture is to popularize why this vaccine can be developed in such a short period and it runs ahead many steps compared to other countries. If these questions could be popularized in a layman-friendly way, I would be more willing to get vaccinated. Because now everyone only knows that the vaccine has been developed and is available for the public, but they do not know how it was developed in fact. (Interview #7)
Topic #6 *Positive perceptions of epidemic situation*	In this area where I live, the epidemic is not very serious, and the protection work is very good. When the epidemic was the worst, people in our area were not infected. After the vaccine is available, it does not matter for many people to get vaccinated. As long as we don’t go out, we will be fine. Many people think so. After all, people hold the opinion that we were all safe in this area when the epidemic was so severe, and now the epidemic has passed for so long, (Interview #3)
	In the case that everyone is vaccinated, he can also be considered safe. That is, if everyone could block the chain of transmission, then he doesn’t need to get vaccinated. (Interview #9)
Topic #7 *Safe and reliable information related to the COVID-19 vaccine*	I think the official media, academicians, such as Nanshan Zhong, and the remarks or appeals made by these pretty authoritative people would have a very positive impression on me. (Interview #1)
	I think it is more customary for me to read detailed parameters and factual reports. I don’t believe it if you simply tell me how safe this thing is. Do not say conclusions, give me data. (Interview #12)

**Table 5:** The key influencing factors derived from two different analysis methods.

	Thematic Analysis	Topic Modeling
**Nonmanipulable factors**	The effectiveness and safety of vaccines	The side effects of the COVID-19 vaccine
	Not found	Application range of vaccine
**Manipulable factors**	Convenience of the vaccine	Potential costs during the vaccination process
	External reference	Influences from others’ experiences
		Potential costs during the vaccination process ^1^
		Safe and reliable information related to the COVID-19 vaccine
	Perceived necessity and urgency of vaccination uptake	Positive perceptions of the epidemic situation
	Scientific and objective expression	Direct and transparent information
	Policy requirements	Not found
	Social responsibility	Not found
	Perceived threat to the freedom of choosing getting vaccinated	Not found
	Perceived benefits	Not found

^1^ This factor appeared twice as its meaning was related to two factors from thematic analysis. Please refer to the following discussion for more explanation.

## Data Availability

The datasets used and analyzed during the current study are available from the corresponding author on reasonable request.

## Appendix A

**Table A1:** 31 Codes generated from the interview transcripts.

No.	Code Name	Operational Definition
1	Vaccine safety	The vaccine does not pose a threat to personal safety, such as a low incidence rate of serious adverse reactions and the good health condition of population after vaccination.
2	Vaccine effectiveness	The effective result and effective time period. That is, the vaccine could protect individuals from the infection of corona virus with a high probability, and the vaccine is effective in a specific period of time.
3	Convenience of vaccine	Costs that need to be considered in the process of vaccination, such as distance and time.
4	The restriction of vaccination uptake	The specific groups that are not suitable for vaccination
5	Introduction of vaccine general information	Basic information about the vaccine, such as the components of the vaccine.
6	Specific explanation of vaccination requirement	Explanation of specific behaviors and requirements in the process of vaccine promotion, such as the specific reasons for people who are not suitable for vaccination
7	Unofficial position	The publisher of the information is not the official medium, but some media with a neutral political position and no political purpose.
8	Official position	The confidence in public power.
9	Scarcity	The scarcer the product, the more people want it. This means that people are more likely to desire or demand products if they are told that they are hard to get.
10	Data exhibition	Provide specific numeric values in the expression.
11	Popular science	Provide vaccine-related scientific information for the public in accurate, rigorous, and easy-to-read language.
12	Social responsibility	People hold the awareness, emotion, and belief that an individual should be responsible for others, family and group, country and society. Moreover, they also have the corresponding conscious attitude that an individual should abide by norms, accept responsibility, and fulfill obligations.
13	Group identification	The individual realizes that he belongs to a particular social group, and at the same time recognizes the emotion and value that group brings to him. This group identification will form a kind of spiritual cohesion, which to some extent regulates the values and moral norms of group members.
14	Risk perception	Individuals’ subjective judgments about how safe their current environment is, how serious the epidemic is, and how much their risk of infection is.
15	Conformity	An individual tends to follow group norms to form beliefs, attitudes, and behaviors.
16	Policy requirement	An individual abandons his or her own opinion and behaves in accordance with policy demands, group regulations.
17	Specializations	Specialization refers to the characteristics of information disseminator, including professional ability, well-trained knowledge, and rich experience.
18	Credibility	Trustworthiness means information receivers believe that the communicator is able and willing to provide objective, fair, true and effective information. Communicator should be sincere, honest and objective, and have no specific communication motives and intentions.
19	Attraction	Attraction means we are more likely to follow a request from someone we admire or like.
20	Dual role persuasion	The information of pros and cons should be both provided.
21	Perceived threat to the freedom of choosing getting vaccinated	Acts with the aim of persuasion. Such acts can be regarded as a threat to freedom if they interfere with individual free choice.
22	Deadline strategy	Increase compliance by indicating to individuals that they have limited time to obtain certain benefits.
23	Vaccination certification	People who take the COVID-19 vaccine will get certification in a certain form, such as the unique health code.
24	Personal involvement degree	Individuals’ subjective experience of the relationship between the personal life and vaccination uptake.
25	Extra benefits	Additional benefits except for vaccine benefits per se.
26	Providing comparison	Provide different quality and different effects of goods for users to compare and choose.
27	Reference group	An individual or group who work as a reference when a person forms an attitude
28	Vicarious experience	Individuals can gain knowledge about vaccination by observing how others behave.
29	In-group pressure	An influence that a group has on its members. When a group member’s thoughts or behaviors conflict with group norms, members feel a mental pressure that they need to abide by group norms in order to maintain their relationship with the group.
30	Certainty of vaccination information	Does not contain arbitrary or ambiguous expressions.
31	Psychological distance	The emotional or psychological distance that an individual maintains with another object.
